# Prevalence and Genetic Analysis of Porcine Circovirus 3 in China From 2019 to 2020

**DOI:** 10.3389/fvets.2021.773912

**Published:** 2021-12-01

**Authors:** Meng Ge, Jie Ren, Yi-Lin Xie, Dun Zhao, Fang-Cheng Fan, Xiao-Qin Song, Man-Xiang Li, Chao-Ting Xiao

**Affiliations:** ^1^College of Veterinary Medicine, Hunan Agricultural University, Changsha, China; ^2^Hunan Engineering Technology Research Center of Veterinary Drugs, College of Veterinary Medicine, Hunan Agricultural University, Changsha, China; ^3^Institute of Pathogen Biology and Immunology, College of Biology, Hunan University, Changsha, China

**Keywords:** China, complete genome, genetic divergence, PCV3, prevalence

## Abstract

Porcine circovirus type 3 (PCV3), a virus belonging to the *Circoviridae* family, is considered to be associated with respiratory and neurological signs, cardiac and multisystemic inflammation, reproductive failure, and porcine dermatitis and nephropathy syndrome-like disease in pigs (*Sus scrofa*). In this study, epidemiological and serological investigations of PCV3 in clinically healthy pigs from different regions of China were performed. Overall, 42.87% (1,101/2,568) of pigs were positive for PCV3 Cap antibody via indirect enzyme-linked immunosorbent assay, with a higher prevalence of PCV3 in multiparous sows (62.22%, 881/1,416) and fattening pigs (28.96%, 159/549) than in suckling piglets (8.96%, 32/357) and nursery pigs (11.79%, 29/246). Of the 2,568 samples, 255 were further tested for PCV3 DNA using real-time polymerase chain reaction, and 63.14% of these were positive, with nearly half having <10 virus copies. The PCV3 DNA and antibody positivity rates were high in the pig serum samples; however, the virus titers and antibody levels were both low, indicating that the humoral immune response of PCV3-infected pigs was weak or lagging, and persistent or repeated infections could occur. Additionally, the complete genomes of 23 PCV3 strains were sequenced and analyzed, which showed nucleotide identities of 98.5~100.0%, 98.6~100.0%, and 99.2~100.0% in the complete genome, open reading frame (ORF)2, and ORF1 sequences, respectively, and amino acid identities of 96.7~100.0% and 99.3~100.0% in the capsid and replicase proteins, respectively. Phylogenetic analysis based on ORF2 nucleotide sequences indicated that the PCV3 strains obtained in the present study could be classified into three sub-clades, with most strains clustered into clade 3c, indicating that PCV3c is the dominant subtype in the regions of China investigated. In general, the present study revealed a high prevalence and high genetic divergence of PCV3 among Chinese pig herds, and indicated that the potential effect of PCV3 on the pig industry may be a concern.

## Introduction

Porcine circoviruses (PCVs), belonging to the genus *Circovirus* of the family *Circoviridae*, are single-stranded circular DNA viruses with a genome of nearly 2 kb (1). PCVs can be divided into four genotypes, namely porcine circovirus 1 (PCV1), PCV2, PCV3, and PCV4 (2, 3). PCV3 infection was first reported in the USA in 2016 by metagenomic sequencing of tissue samples from pigs (*Sus scrofa*) with porcine dermatitis and nephropathy syndrome (PDNS), reproductive failure, myocarditis, and multi-systemic inflammation (4, 5). Further studies confirmed that PCV3 is associated with reproductive failure, enteric disease, and central nervous system symptoms, including congenital tremors and systemic inflammatory disease (6), indicating that PCV3, like PCV2, could be an important pathogen in the pig industry.

PCV3 has a 2,000 bp long circular genome, containing three open reading frames (ORFs): ORF1, ORF2, and ORF3. ORF1, and ORF2 encode the replicase (Rep) and capsid (Cap) proteins, respectively. The function of the protein encoded by ORF3 has not yet been described (5). The Cap protein plays an important role in the antigenicity of circoviruses, including PCV2 and PCV3 (7), and PCV3 strains circulating worldwide can be divided into two subtypes (PCV3a and PCV3b) based on the amino acid sequence of the Cap protein (8–10).

PCV3 has recently been identified worldwide, including in Asia, Europe, and South and North America (11). In China, PCV3 infection in pigs has been reported in more than 20 provinces and regions (12). Other than in pigs, PCV3 nucleic acids have been detected in other species in China, such as cattle (*Bos taurus*) (13), dogs (*Canis lupus*) (14), and several species of mosquitoes (15). The occurrence of PCV3 infection was initially documented in America, but a retrospective study revealed that it could be traced back to 1996 in China and Spain, indicating that PCV3 has been prevalent among pig populations for more than 20 years (16, 17).

In the present study, in order to investigate the prevalence of PCV3 in China, an indirect enzyme-linked immunosorbent assay (ELISA) was performed to test 2568 serum samples, randomly collected from clinically healthy pigs from 17 provinces of China between 2019 and 2020; 255 serum samples were chosen to detect the rate of PCV3 DNA; moreover, to further investigate the genetic divergence of PCV3 in China, the complete genomes of 23 PCV3 strains from different regions were sequenced and analyzed.

## Materials and Methods

### Serum Sample Collection

Serum samples from 2,568 clinically healthy pigs from 36 large-scale pig farms were collected from 17 provinces (Hunan, Xinjiang, Jilin, Zhejiang, Jiangsu, Jiangxi, Guangxi, Hebei, Shandong, Shanxi, Anhui, Yunnan, Hubei, Inner Mongolia, Henan, Sichuan, and Guizhou) between 2019 and 2020. The criteria for specimens collection are as follows: (1) covering different age groups including suckling pigs, nursery pigs, grow-finishing pigs and multiparous sows; (2) only clinically healthy pigs collected; (3) up to ten samples, if possible, collected from each age group in a farm. The specimens were collected from each farm using standard procedures and were delivered to the Laboratory of Molecular Biology and Immunology, Hunan Agricultural University. Detailed information on the specimens from different provinces and age groups is presented in Tables 1, 2.

**Table 1:** Seroprevalence of PCV3 in serum samples from different provinces of China by ELISA.

**Sample region**	**Specimen numbers**	**Positive samples**	**Positive rate (%)**
Xinjiang	135	22	16.30
Henan	144	36	24.56
Sichuan	90	3	3.33
Inner Mongolia	120	47	39.17
Jilin	137	43	31.39
Zhejiang	60	31	51.67
Jiangsu	59	31	52.54
Jiangxi	62	11	19.35
Guangxi	91	6	6.59
Hebei	60	36	60.0
Shandong	59	31	52.54
Shanxi	58	2	3.45
Anhui	113	47	41.59
Yunana	72	18	25.00
Hubei	60	27	45.00
Guizhou	73	21	28.77
Hunan	1,175	689	58.64
Total	2,568	1,101	42.87

*PCV3, porcine circovirus type 3; ELISA, enzyme-linked immunosorbent assay*.

**Table 2:** Seroprevalence of PCV3 in different age groups in China by ELISA.

**Age group**	**No. of specimens**	**No. of positive**	**Positive rate (%)**
Sucking piglets	357	32	8.96
Nursery pigs	246	29	11.79
Grow-finish pigs	549	159	28.96
Multiparous sows	1,416	881	62.22
Total	2,568	1,101	42.87

*PCV3, porcine circovirus type 3; ELISA, enzyme-linked immunosorbent assay*.

### Indirect ELISA

Blood samples were centrifuged at 2,500 × g for 15 min, and the supernatants were collected for PCV3 Cap-specific antibody detection via the ELISA method established in our laboratory (18). Briefly, high-binding 96-well microtitration plates were coated with the purified PCV3 Cap protein (expressed in *Escherichia coli* cells and the predicted nuclear localization sequence, NLS, 33 amino acids from amino terminal of Cap protein was excluded) in 0.05 M NaHCO_3_ buffer (pH 10.6), incubated overnight at 4°C, and then blocked with phosphate-buffered saline/Tween (PBST) containing 5% skimmed milk. One hundred microliters of the serum samples diluted with PBST containing 5% skimmed milk were added and incubated at 37°C for 1 h, then the plates were washed four times with PBST. Next, 100 μl of horseradish peroxidase-conjugated goat anti-swine IgG diluted antibodies (KPL, Gaithersburg, MD, USA) were added to each well and incubated at 37°C for 30 min, then the plates were washed four times with 100 μl PBST. Finally, the peroxidase reaction was visualized using tetramethylbenzidine-hydrogen peroxide solution as the substrate (KPL, Gaithersburg, MD, USA). The reaction was terminated by adding 50 μl of 2 M sulfuric acid to each well, and the optical density at 450 nm of the plates was read using a microplate reader (FC; Thermo Lab system, Helsinki, inland).

### Detection of PCV3 in the Sera by Real-Time Polymerase Chain Reaction

Among the 2,568 serum samples, 255 (6–12 from each farm) were randomly chosen for nucleic acid extraction using a commercial kit (Bioer, Hangzhou, China), and were then used to investigate the PCV3 prevalence by real-time polymerase chain reaction (q-PCR) following the protocols described previously (19).

### PCV3 Complete Genome Sequencing

To investigate the genetic divergence of PCV3 in China, samples with a Ct value <35 were used to amplify the complete genome of the virus with two pairs of primers (Table 3). The PCR reactions (50 μL) were performed under the following conditions: 25 μL 2 × Taq Master mix (Takara Biotechnology, Dalian, China), 1.0 μL of each primer, 4.0 μL of DNA template, and 19 μL of sterilized water. The PCR conditions were 95°C for 5 min, 35 cycles at 95°C for 30 s, 58°C for 30 s, and 72°C for 1 min, followed by final elongation at 72°C for 10 min. The PCR products were purified and cloned into the pMD19-T vector for sequencing, and the obtained sequences were submitted to the GenBank database (Table 4).

**Table 3:** Primers used in this study to amplify the complete genome of PCV3.

**Primer**	**Sequence**	**Primer position**	**Length**
AF	CGGAGGGAAAGCCCGAAAC	219–237	1,561
AR	CGCCTAAACGAATGGGAAACT	1,759–1,779	
BF	TTTCCGCATAAGGGTCGTCTT	1,596–1,616	1,020
BR	CAGGCATCTTCTCCGCAACT	596–615	

*PCV3, porcine circovirus type 3*.

**Table 4:** Detailed information on the PCV3 strains identified in the present study, including strain name, country, collection date, host, and GenBank accession number.

**Strains**	**Province**	**Collection year**	**Accession number**
PCV3/China-Hunan-Zhuzhou/2020	Hunan	2020	MW855574
PCV3/China-Hunan-Changsha/2020	Hunan	2020	MW855575
PCV3/China-Hunan-Leiyang/2019	Hunan	2019	MW855576
PCV3/China-Hunan-Fenghuang/2020	Hunan	2020	MW855577
PCV3/China-Hunan-Chengzhou/2019	Hunan	2019	MW855578
PCV3/China-Hunan-Yueyang/2019	Hunan	2019	MW855579
PCV3/China-Hunan-Yongzhou/2019	Hunan	2019	MW883346
PCV3/China-Hunan-Yiyang/2020	Hunan	2020	MW883347
PCV3/China-Hunan-Shaoyang/2019	Hunan	2019	MW883348
PCV3/China-Hunan-Huaihua/2019	Hunan	2019	MW883349
PCV3/China-Hunan-Changde/2019	Hunan	2019	MW883350
PCV3/China-Guizhou/2020	Guizhou	2020	MZ449237
PCV3/China-Hebei/2020	Hebei	2020	MZ449238
PCV3/China-Henan/2020	Henan	2020	MZ449239
PCV3/China-Shanxi/2020	Shanxi	2020	MZ449243
PCV3/China-Inner Mongolia/2020	Inner Mongolia	2020	MZ449242
PCV3/China-Sichuan/2020	Sichuan	2020	MZ449244
PCV3/China-Xinjiang/2020	Xinjiang	2020	MZ449245
PCV3/China-Yunnan/2020	Yunnan	2020	MZ449246
PCV3/China-Zhejiang/2020	Zhejiang	2020	MZ449247
PCV3/China-Jiangxi/2020	Jiangxi	2020	MZ449241
PCV3/China-Jilin/2020	Jilin	2020	MZ449240
PCV3/China-Anhui/2020	Anhui	2020	MZ449236

*PCV3, porcine circovirus type 3*.

### Bioinformatics Analyses

To further analyze the genetic characteristics of the PCV3 strains, the complete PCV3 sequences obtained in the present study and the reference strains (*n* = 15) downloaded from GenBank were aligned using ClustalX and Lasergene software for homology analysis. A phylogenetic tree was generated using the neighbor-joining method in MEGA 6.0, with 1,000 bootstrap replicates (20).

### Statistical Analysis

Statistical analyses were performed using SPSS V20.0 (IBM, Chicago, IL). The differences were considered statistically significant when *p* < 0.05.

## Results

### PCV3 Seroprevalence in China

PCV3 Cap antibodies could be detected in 1,101 out of 2,568 serum samples, with a positive rate of 42.87, and 91.67% (33/36) of the pig farms were PCV3-positive (Table 1), with the positive rates of different provinces varying from 3.33 to 60.0%. Additionally, the correlation between PCV3 seroprevalence and pig age groups was analyzed, the results showing that the highest seroprevalence of PCV3 was observed in multiparous sows (62.22%, 881/1,416), significantly higher (*p* < 0.05) than that of fattening pigs (28.96%, 159/549), nursery pigs (11.79%, 29/246), and suckling piglets (8.96%, 32/357; Table 2).

### PCV3 Positive Rate in the Serum Samples

According to the standard curve for the qPCR assay, which were determined by serial 10-fold dilutions of the plasmid DNA of PCV3 ORF1 gene, the samples with cycle threshold (Ct) ≤ 40 (Ct = 40 corresponding to 1 virus copy in 1 μL sample DNA) were regarded as PCV3 positive. Of the 255 samples, 161 (63.14%) were PCV3 PCR positive, with the rates of 45.71% (32/70), 74.68% (59/79), and 66.04% (70/106) detected in nursery pigs, grow-finishing pigs, and sows, respectively (Table 5). However, the Ct values of 47.2% (76/161) of the positive samples were higher than 35, indicating that the viral copy number in these samples was <10 copies/μL (data not shown).

**Table 5:** The PCV3 DNA positive rates in serum samples of different age groups investigated by real-time PCR (qPCR) and the distribution of qPCR positive samples.

**Age group**	**No. of specimens**	**qPCR**		**ELISA result**		**Distribution of qPCR positive samples**	
		**No. of positive**	**Positive rate (%)**	**No. of positive**	**Positive rate (%)**	**Numbers in ELISA negative samples**	**Numbers in ELISA positive samples**
Nursery pigs	70	32	45.71	10	14.29	23/32(71.88%)	9/32(28.12%)
Grow-finish pigs	79	59	74.68	28	35.44	37/59(62.71%)	22/59(37.29%)
Multiparous sows	106	70	66.04	76	71.70	17/70(24.29%)	53/70(75.71%)
Total	255	161	63.14	114	44.71	77/161(47.83%)	84/161(52.17%)

*PCV3, porcine circovirus type 3; PCR, polymerase chain reaction*.

Furthermore, 44.71% (114/255) of the above 255 samples were seropositive, with the highest (71.7%) and the lowest (14.29%) observed in sows and nursery pigs, respectively, and a seropositive rate of 35.44% detected in grow-finish pigs (Table 5), which is similar to the overall serologic results based on the 2,568 serum samples. Most of the PCV3 DNAs were detected in PCV3 seropositve samples, while there are also many PCV3 DNAs were detected in PCV3 seronegative samples with a high rate of 71.88% (23/32) in nursery pigs, and 62.71% (37/59) in grow-finish pigs (Table 5).

### Genome Sequence Analysis

To analyze the genetic characteristics of PCV3 strains in China, 23 complete genome sequences of PCV3 strains obtained from different regions in China were successfully amplified and sequenced, and all the obtained sequences were submitted to GenBank (Table 4). The complete genomes of the 23 PCV3 strains, 2,000 nucleotides in length, showed sequence identities of 98.5~100.0, 98.6~100.0, and 99.2~100.0% in the complete genome, ORF2, and ORF1, respectively, and 96.7~100.0 and 99.3~100.0% amino acid similarity in the Cap and Rep proteins, respectively (Table 6). Moreover, the PCV3 genome sequences identified in this study displayed nucleotide identities of 96.9~99.5, 98.9~99.8, and 98.3~99.9% in ORF2, ORF1, and the complete genome, respectively, and amino acid identities of 95.3~100.0 and 99.2~100.0% in the Cap and Rep proteins, respectively, compared to other PCV3 reference strains (Table 6). Additionally, PCV3 strains showed <60 and 50% nucleotide and amino acid homologies, respectively, with PCV1, PCV2, and PCV4 representative strains (Table 6).

**Table 6:** Sequence homology analysis of the PCV3 strains identified in the present study.

**Selected strains**	**Nucleotide**			**Amino acid**	
	**ORF1 (%)**	**ORF2 (%)**	**Complete sequence (%)**	**ORF1 (%)**	**ORF2 (%)**
PCV3 strains identified in this study	99.2~100.0	98.6~100.0	98.5~100.0	99.3~100.0	96.7~100.0
Compared with other PCV3 strains	98.9~99.8	96.9~99.5	98.3~99.9	99.2~100.0	95.3~100.0
Compared with PCV1 strains	59.7~50.1	43.4~44.1	43.5~44.0	45.5~45.9	24.4~25.2
Compared with PCV2 strains	50.3~50.9	46.1~46.5	42.7~43.1	46.3~47.4	25.9~27.8
Compared with PCV4 strains	53.4~53.8	43.9~44.6	43.6~45.0	48.6~49.7	23.2~23.9

*PCV3, porcine circovirus type 3*.

### Amino Acid Sequence Alignments

Amino acid sequence alignments among the Cap and Rep proteins of the 23 PCV3 strains obtained in this study and 17 reference PCV3 strains from the GenBank database were performed, and no amino acid deletions or insertions were observed among the 23 strains identified in this study, while a total of 18 and 3 amino acid substitutions were observed in Cap and Rep (Supplementary Table 1), respectively. These were mainly located at sites 5, 10, 24, 110, 124, 137, and 152 in Cap; and 55, 122, and 286 in Rep. Interestingly, a series of unique amino acid substitutions were observed in the Cap of PCV3 isolates from the city of Leiyang in the Hunan province, such as the substitutions at sites 5 (A → P), 10 (P → R), 115 (W → R), and 180 (V → A). The significance of these amino acid substitutions needs further study.

### Phylogenetic Analysis

**Figure 1:**
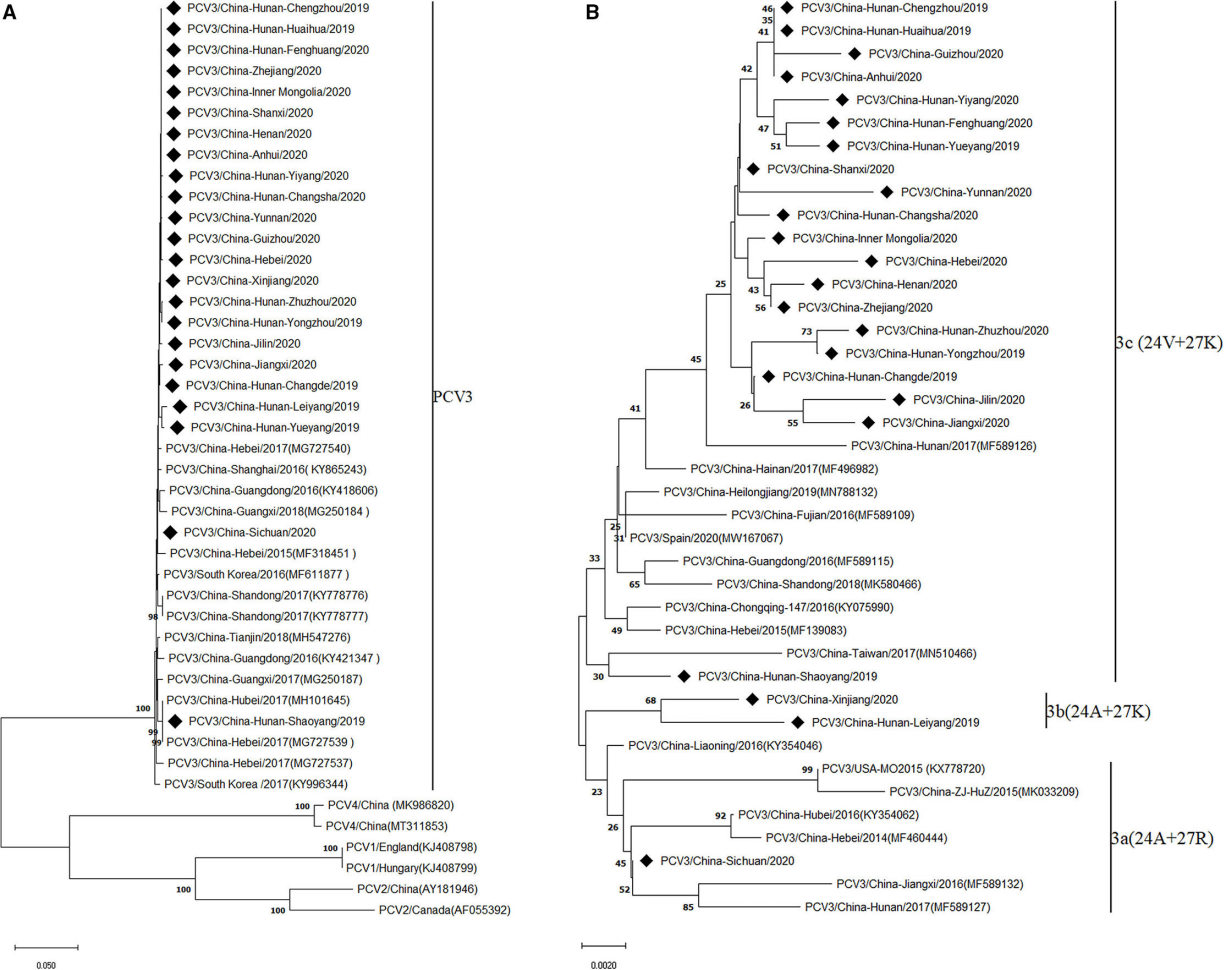
Phylogenetic analysis of the complete genome **(A)** and capsid protein **(B)** of 23 PCV3 strains obtained in this study and reference strains from GenBank. Phylogenetic trees were constructed by the neighbor-joining method using MEGA 6.0 software. Black squares represent PCV3 strains identified in this study.

To investigate the evolutionary relationships between the PCV3 strains obtained in the present study and other PCV3 strains, phylogenetic analysis was performed based on the complete genomes of the 23 PCV3 strains and the reference strains. The results showed that all PCV3 strains clustered into a monophyletic clade, which showed a closer phylogenetic relationship with PCV4 strains, but a larger distance from PCV1 and PCV2 strains (Figure 1A), in line with previous conclusions (7, 21). Considering the high genetic variation of the ORF2 gene compared with ORF1 and the complete genome, it has been proposed to classify PCV3 strains into three sub-clades (PCV3a, PCV3b, and PCV3c) based on the evolutionary characteristics of the Cap protein (7, 22). Similarly, based on the two amino acid mutations (A24V and R27K) in the Cap protein, the PCV3 strains investigated in the present study were divided into three clades (Figure 1B), with most strains (86.96%, 20/23) belonging to group 3c, and two and one strains clustered into groups 3b and 3a, respectively, which indicated that the prevalence of PCV3c increased during PCV3 evolution.

## Discussion

PCV3 is a newly emerging porcine viral pathogen considered to be associated with different clinical presentations in pigs, such as PDNS, reproductive failure, and multi-systemic inflammation (5, 22, 23). Since the first identification of PCV3 in the USA in 2016, growing evidence indicates that this pathogen has widely spread in pig populations (11). To date, the PCV3 strains prevalent worldwide have been divided into two genotypes (PCV3a and PCV3b) based on the genetic characteristics of PCV3 complete genome, and the prevalence of PCV3a has recently increased (24). Considering the fact that there are no available vaccines against PCV3, obtaining accurate information on the prevalence and genetic characteristics of PCV3 circulating in China will provide scientific data for developing effective measures against this infectious disease.

In this study, by investigating the seroprevalence of PCV3 in healthy pigs from 2019 to 2020 in China, several characteristics of this virus could be summarized: (1) an average PCV3-seroprevalence of 42.87% (1,101/2,568) was observed in 91.67% (33/36) of the pig farms, which is consistent with recent reports (25, 26) and suggests a high prevalence of PCV3 in China; (2) the seropositivity rate of PCV3 in different provinces ranged from 3.33 to 60.0%, and the relatively low seroprevalence of PCV3 observed in the Sichuan (3.33%, 3/90), Shanxi (3.45%, 2/58), and Guangxi (6.59%, 6/91) provinces might be due to the limited sample size; (3) the rate of PCV3-positive sera in multiparous sows (61.85%, 634/1,025) and fattening pigs (24.37%, 223/915) was higher than in nursery pigs (12.64%, 46/364) and suckling piglets (9.38%, 57/608), indicating limited maternal antibody in suckling pigs, which was consistent with a previous study (25). Furthermore, the *S*/*P* values of the positive specimens were low (most of them were <0.5), suggesting a low humoral immune response induced by PCV3 infection in pigs, which may be related to the limited antigen concentrations in the sera, since the serum specimens were collected from pigs with no obvious symptoms (4, 25).

Real-time PCR showed a high PCV3 prevalence of 63.14% (161/255) in China, and the Ct values of almost half of the positive samples were above 35, indicating low viral titers in the sera, which confirmed the presumption from the above ELISA results that the concentration of viral antigen was low in most serum samples. A high prevalence of PCV3 has also been reported in the Zhejiang Province, China, with an overall PCV3 positive rate of 67.1% from 283 clinical samples taken from 2014 to 2017 (27). However, the present positive rate is much higher than the PCV3 prevalence of 8% (52/624) observed in European pig herds (28), which may be related to geographic differences and feeding environments. Moreover, the PCV3 DNA positive rates were much higher than the PCV3 antibody-positive rates in nursery and grow-finishing pigs, which may be related to the low viral titers in the majority of the serum samples.

Interestingly, the PCV3 positive rate in sows was still high, with the majority of them also being seropositive (53/70), suggesting that low-level PCV3 antibody showed very limited effect on neutralizing or clearing up the virus. This will probably lead to persistent or repeated infection in sows, and in turn to the high prevalence of PCV3 in pig sera.

Overall, based on the present PCV3 serological and DNA detecting results, we further proposed that the low viral titers in the sera is the result of low replication level of PCV3 virus in these clinically healthy pigs, which may lead to low level of immune reaction or lagging immune reaction, although the pigs got infected during early age period like in nursery or fattening state, and then this may help the virus to achieve persistent infection in pigs, otherwise a high antibody level would lead to the virus to be cleared up soon. Thus based on these points, the relatively low antibody levels but a rather high PCV3 DNA positive rates in nursery and grow-finishing pigs could be explained, and also for the highest antibody positive rate but with low *S*/*P* values in multiparous sows observed in the present study.

To further analyze the genetic characteristics and evolution of the PCV3 strains prevalent in China, the complete genome sequences of 23 PCV3 strains obtained in this study were amplified, sequenced, and analyzed. The results showed that these strains shared more than 96% identity at the nucleotide and amino acid sequence levels, indicating that the genome of PCV3 strains circulating in China remains highly conserved, in line with previous research (24, 29).

The Cap protein encoded by the ORF2 gene is the major structural protein that mainly induces the host immune response in circoviruses (30). Therefore, investigating amino acid mutations in this protein can improve our understanding of PCV3 evolution. In this study, a series of amino acid mutations were observed in the Cap protein, mainly occurring at positions 5 (A–P), 10 (R–K), 24 (V–A), 110 (I–M), 124 (D–G), 137 (S–F), and 152 (A–G) (Supplementary Table 1). Li et al. reported that the frequency of the amino acid mutation A to V at position 24 of the Cap protein gradually increased during 2015–2018, but remained <50% (24). In this study, the frequency of the mutation 24 (A → V) reached nearly 90% (20/23), indicating a high amino acid substitution rate at this position. Given that variations in key amino acid sites of Cap protein would affect the viral virulence or the pathogenicity of circoviruses (31), further experimental studies should be carried out to investigate the roles of these mutations in PCV3 strains, including immunogenicity of the Cap protein, virus replication, and virulence.

Growing evidence suggests that two amino acid mutations (A24V and R27K) in the Cap protein could be employed as molecular markers to analyze the evolutionary relationship of PCV3 strains (7, 32). A phylogenetic tree based on the Cap protein of PCV3 strains showed that PCV3 isolates could be divided into three sub-clades (PCV3a, PCV3b, and PCV3c) according to the amino acid substitutions at positions 24 and 27 of the Cap protein. In our study, 86.96% (20/23) of PCV3 isolates were PCV3c strains, a much higher proportion than that reported in previous studies (24, 32). Qi et al. reported that only 29.4% (15/51) of PCV3 isolates from 27 provinces of China from 2015 to 2017 were classified as PCV3c strains (32). However, two problems need to be further investigated: first, the pathogenic characteristics of PCV3 strains from different phylogenetic clades; and second, the factors contributing to the rapid molecular evolution of PCV3, since a commercial vaccine against PCV3 is currently unavailable, indicating an absence of immune pressure due to vaccine usage.

In summary, in the present study the prevalence of PCV3 in different provinces of China and the genetic divergence of the complete genomes of PCV3 strains were analyzed. The results indicated a high prevalence of PCV3 in the Chinese pig population and a high amino acid variant rate in the Cap protein, suggesting that further studies should be performed to clarify the influence of these amino acid mutations on the pathogenic characteristics of PCV3.

## Data Availability Statement

The datasets presented in this study can be found in online repositories. The names of the repository/repositories and accession number(s) can be found in the article/Supplementary Material.

## Ethics Statement

The animal study was reviewed and approved by Animal Ethics Committee of Hunan Agricultural University, Hunan, China. Written informed consent was obtained from the owners for the participation of their animals in this study.

## Author Contributions

M-XL, C-TX, and MG conceived and designed the experiments. JR, Y-LX, DZ, X-QS, and F-CF performed the experiments. M-XL, MG, JR, and C-TX analyzed the data. JR, C-TX, and MG wrote the paper. All authors contributed to the article and approved the submitted version.

## Funding

This work was supported by the National Natural Science Foundation of China (Grant No. 32072871) and the Natural Science Foundation of Hunan Province, China (Grant No. 2019JJ50258).

## Conflict of Interest

The authors declare that the research was conducted in the absence of any commercial or financial relationships that could be construed as a potential conflict of interest.

## Publisher's Note

All claims expressed in this article are solely those of the authors and do not necessarily represent those of their affiliated organizations, or those of the publisher, the editors and the reviewers. Any product that may be evaluated in this article, or claim that may be made by its manufacturer, is not guaranteed or endorsed by the publisher.
