# Supplementary material for: Prevalence and Genetic Analysis of Porcine Circovirus 3 in China From 2019 to 2020
Source: Front Vet Sci. 2021 Dec 1;8:773912. doi: 10.3389/fvets.2021.773912 (PMC8671461; doi:10.3389/fvets.2021.773912)
Supplement: Supplementary file 1 [file Table_1.DOCX]

|  | **Cap** | | | | | | | | | | | | | | | | | | | **Rep** | | |
| --- | --- | --- | --- | --- | --- | --- | --- | --- | --- | --- | --- | --- | --- | --- | --- | --- | --- | --- | --- | --- | --- | --- |
|  | 5 | 10 | 24 | 26 | 27 | 95 | 110 | 115 | 124 | 137 | 152 | 168 | 178 | 180 | 182 | | 202 | 203 | 205 | 57 | 122 | 286 |
| PCV3-Xinjiang | P | K | A | R | K | S | I | W | D | S | A | R | P | V | W | | G | T | E | K | A | K |
| PCV3-Anhui | A | R | V | R | K | S | I | W | D | S | A | R | P | V | W | | G | T | E | K | S | K |
| PCV3-Inner | A | R | V | R | K | S | I | W | D | S | A | R | P | V | W | | G | T | E | K | S | K |
| PCV3-Changde | A | R | V | R | K | S | I | W | D | S | A | R | P | V | W | | G | T | E | K | A | K |
| PCV3-Huaihua | A | R | V | R | K | S | I | W | D | S | A | R | P | V | W | | G | T | E | K | A | K |
| PCV3-Shanxi | A | R | V | R | K | S | I | W | D | S | A | R | P | V | W | | G | T | E | K | A | K |
| PCV3-Zhejiang | A | R | V | R | K | S | I | W | D | S | A | R | P | V | W | | G | T | E | K | A | K |
| PCV3-Chengzhou | A | R | V | R | K | S | I | W | D | S | A | R | P | V | W | | G | T | E | K | A | K |
| PCV3-Henan | A | K | V | R | K | S | I | W | D | S | A | R | P | V | W | | G | T | E | K | A | K |
| PCV3-Changsha | A | R | V | R | K | S | M | W | D | S | A | R | P | V | W | | G | T | E | K | A | K |
| PCV3-Jiangxi | A | R | V | R | K | S | I | W | D | S | A | R | P | V | W | | A | T | E | K | S | K |
| PCV3-Yueyang | A | R | V | R | K | S | I | W | D | S | G | K | P | V | W | | G | T | E | K | A | K |
| PCV3-Yiyang | A | R | V | R | K | S | I | W | D | S | G | R | P | V | W | | G | T | K | K | A | N |
| PCV3-Sichuan | A | R | A | R | R | S | I | W | D | S | A | R | P | V | W | | G | T | E | K | S | K |
| PCV3-Zhuzhou | A | R | V | R | K | S | M | W | G | S | A | R | P | V | W | G | | T | E | K | A | K |
| PCV3-Yongzhou | A | R | V | R | K | S | I | W | G | S | A | R | P | V | W | | G | T | E | K | A | K |
| PCV3-Hebei | A | R | V | R | K | S | I | W | D | S | A | R | P | V | W | | G | T | E | K | A | K |
| PCV3-Jilin | A | R | V | R | K | S | I | W | D | F | A | R | P | V | W | | G | P | E | R | S | K |
| PCV3-Yunan | A | R | V | R | K | S | I | W | D | F | A | R | P | V | W | | G | T | E | K | A | K |
| PCV3-Fenghuang | A | R | V | R | K | S | I | W | D | S | G | R | S | V | W | | G | T | E | K | A | K |
| PCV3-Guizhou | A | R | V | R | K | F | I | W | D | S | A | R | P | V | G | | G | T | E | K | S | K |
| PCV3-Leiyang | P | K | A | G | K | S | I | R | D | S | A | R | P | A | W | | G | T | E | R | S | N |
| PCV3-Shaoyang | A | R | V | R | K | S | I | W | D | S | A | R | P | V | W | | G | T | E | K | A | K |

**Supplemental Table 1.** **Comparison of Cap and Rep amino acid sequences of PCV3 strains identified in the present study**
